# Serum/glucose starvation enhances binding of miR-4745-5p and miR-6798-5p to *HNRNPA1* mRNA 3ʹUTR: A novel method to identify miRNAs binding to mRNA 3ʹUTR using λN peptide-boxB sequence

**DOI:** 10.1016/j.ncrna.2025.01.001

**Published:** 2025-01-07

**Authors:** Tetsuyuki Takahashi, Mai Funamura, Shun Wakai, Takao Hijikata

**Affiliations:** Department of Anatomy and Cell Biology, Faculty of Pharmacy, Research Institute of Pharmaceutical Sciences, Musashino University, Nishi-Tokyo, Tokyo, 202-8585, Japan

**Keywords:** microRNA, hnRNP A1, λN/boxB system, Serum/glucose starvation, Microarray, miR-4745-5p, miR-6798-5p

## Abstract

Serum/glucose starvation causes complete loss of heterogeneous nuclear ribonucleoprotein A1 (hnRNP A1) without altering mRNA levels. However, the mechanisms driving hnRNP A1 downregulation during serum/glucose starvation are not yet well understood. Using the novel interaction between the λN peptide and boxB sequence (λN/boxB system) and miRNA microarray analysis, we aimed to identify specific-binding microRNAs (miRs or miRNAs) targeting *HNRNPA1* mRNA 3ʹUTR under serum/glucose-starved conditions. Four miRNAs were identified as serum/glucose starvation-driven miRNAs for *HNRNPA1* mRNA 3ʹUTR. Reporter assays, anti-miRNA and mutated miRNA-based assays, photoactivatable ribonucleoside-enhanced crosslinking and immunoprecipitation/reverse transcribed-quantitative polymerase chain reaction, and transient overexpression of miRNAs showed that miR-4745-5p and miR-6798-5p suppress hnRNP A1 protein levels via enhancement of binding to *HNRNPA1* mRNA 3ʹUTR under serum/glucose-starved condition. miR-4745-5p and miR-6798-5p overexpression significantly decreased growth rates, which was rescued by co-transfection with anti-miRNA for miR-4745-5p and miR-6798-5p. Anti-miRNA transfection for miR-4745-5p and miR-6798-5p significantly increased growth rates under serum/glucose-starved conditions. Furthermore, hnRNP A1 overexpression recovered miR-4745-5p- and miR-6798-5p-induced growth suppression. These findings indicated that miR-4745-5p and miR-6798-5p are serum/glucose starvation-driven miRNAs for hnRNP A1 and validated the λN/boxB system as a simple and useful method for detecting mRNA 3ʹUTR-bound miRNA.

## 1 Introduction

Heterogeneous nuclear ribonucleoprotein A1 (hnRNP A1) is an RNA-binding protein involved in mRNA splicing, transport, stability, transcriptional and translational regulation, and telomere maintenance via specific binding to the target sequence [[1], [2], [3], [4], [5], [6], [7]]. These functions of hnRNP A1 contribute to cellular homeostasis and protect normal movements of RNA in cells. Overexpression or dysfunction of hnRNP A1 is found in lesions of diseases, including neurodegenerative disorders and cancers [3,[8], [9], [10], [11], [12], [13], [14], [15], [16], [17]]. We previously reported that serum/glucose-starved conditions, which mimics undernutrition in lesions of incomplete blood supply (e.g., within the core of tumors or lesions of cerebral and myocardial infarction), leads to a complete loss of hnRNP A1 without any corresponding changes in mRNA levels in HeLa cervical cancer cells [18]; however, the definitive molecular mechanism of this downregulation in serum/glucose-starved conditions remains unknown.

We hypothesized that translational suppression participates in this process. miRNA, recognized as a key translational regulator [19], binds to the 3ʹUTR of target mRNA via specific nucleotide sequences (seed sequences), and cellular stresses, including serum starvation, can alter of particular miRNA levels, reducing specific protein levels [[20], [21], [22], [23], [24]].

To detect miRNAs involved in hnRNP A1 loss under serum/glucose-starved conditions, we established a novel miRNA detection system, λN/boxB. This λN/boxB system uses a pulldown method with cell lysates that co-expresses a tag protein-fused λN peptide and boxB sequence-fused RNA (This system is customizable, allowing design of specific tag proteins and RNA sequences as needed). This study aimed to identify miRNAs that contribute to the loss of hnRNP A1 protein under serum/glucose-starved conditions and investigate their effects on cellular growth.

## 2 Materials and methods

### 2.1 Cells

Human cervical cancer HeLa cells were maintained in DMEM (Wako, Osaka, Japan) supplemented with 10 % fetal bovine serum (Sigma-Aldrich, St. Louis, MO, USA), 100 units/mL of penicillin G (Wako), and 0.1 mg/mL of streptomycin sulfate (Wako).

### 2.2 Plasmids

This study used various mammalian expression plasmids including pZac, pZac-5 × boxB, pZac-RNP3ʹUTR-5 × boxB, pZac-RNP3ʹUTR/miR-4734mt-5 × boxB, pZac-RNP3ʹUTR/miR-4745mt-5 × boxB, pZac-RNP3ʹUTR/miR-6126mt-5 × boxB, pZac-RNP3ʹUTR/miR-6798mt-5 × boxB, pQCXIP, pQCXIP-RNPA1-HA, which is not inserted the *HNRNPA1* mRNA 3ʹUTR, and the λN peptide-fused Halo-Tag expression plasmid pFC-λN-HA-Halo. Additionally, then luciferase reporter plasmids included pGL, pRL-CMV, pRL-Luc-RNP3ʹUTR, pRL-Luc-RNP3ʹUTR/miR-4734mt, pRL-Luc-RNP3ʹUTR/miR-4745mt, pRL-Luc-RNP3ʹUTR/miR-6126mt, and pRL-Luc-RNP3ʹUTR/miR-6798mt. Retroviral miRNA expression plasmids included pSIREN-RetroQ (pSRQ), pSRQ-miR4734, pSRQ-miR4745, pSRQ-miR6126, and pSRQ-miR6798. The procedure of plasmid construction and primer sequences are provided in the supporting information and Supplementary Table 1, respectively.

### 2.3 Serum/glucose starvation

Serum/glucose starvation was performed by replacing the medium with to serum-free glucose-deprived DMEM (Wako) after washing the cells twice with phosphate-buffered saline. The glucose-deprived DMEM was changed every 3 weeks to maintain the condition as fresh condition.

### 2.4 Reverse transcription-polymerase chain reaction (RT-PCR)

Total RNA (500 ng/reaction) from transfected cells was reverse transcribed using a random hexamer and ReverTra Ace (TOYOBO, Osaka, Japan) following the manufacturer's protocol. Semiquantitative RT-PCR was conducted with cDNAs obtained from reverse transcription using PrimeStar GXL (Takara Bio Japan, Shiga, Japan) for pZac-based vector-derived RNAs and actin mRNA (an internal control). The thermocycling program included initial denaturation at 94 °C for 2 min; 30 cycles of amplification at 98 °C for 10 s, 60 °C for 15 s, and 68 °C for 3 min; and a final extension at 68 °C for 10 min. RT-PCR products were separated using 1.5 % agarose gel electrophoresis and were subsequently visualized using a UV transilluminator. The primer sequences used in this experiment are listed in Supplementary Table 1.

### 2.5 Immunoblotting

Cells were lysed with 2 × SDS sample buffer (50 mM Tris-HCl at pH 6.8, 10 % glycerol, 2 % SDS, 0.1 % bromophenol blue, and 0.2 M dithiothreitol [DTT]) and a Bio-Rad Protein Assay Kit (Bio-Rad, Hercules, CA, USA) was used to quantify sample protein concentrations. Subsequently, cell lysates (12 μg of protein) were subjected to SDS-PAGE (10 % gel) and transferred to polyvinylidene fluoride membranes. The ECL-Prime Western Blotting Detection Reagent (GE Healthcare UK, Buckinghamshire, UK) was used to detect immunoreactive signals. Antibodies used in this study are listed in the supporting information. A mouse antibody against HaloTag (1:1000; G9211, Promega, Madison, WI, USA), rabbit antibody against hnRNP A1 (1:2000; GTX106208, GeneTex, Alton Parkway Irvine, CA, USA), and mouse antibody against β-actin (1:5000; clone AC-15, Sigma-Aldrich) were used as primary antibodies. Goat anti-rabbit IgG-HRP and goat anti-mouse IgG-HRP (both at 1:5000; Sigma-Aldrich) were used as secondary antibodies.

### 2.6 Isolation of HNRNPA1 mRNA 3ʹUTR-bound RNAs via the λN/boxB system

Cells (2 × 10^6^) were seeded into 100-mm dishes and preincubated for 16 h at 37 °C. Thereafter, cells were co-transfected with 5 × boxB-fused RNA- and Halo tag-fused λN-expression vectors using polyethyleneimine MAX (Polysciences, Warrington, PA, USA). Cells were subsequently lysed with avidin-pulldown lysis buffer (20 mM Tris-HCl pH 7.4, 100 mM KCl, 5 mM MgCl_2_, 0.5 % NP-40, and 0.5 mM DTT) supplemented with RNaseOUT (Invitrogen, Carlsbad, CA, USA) and pulled down with HaloLink™ Resin (Promega) for 1 h at 25 °C. RNAs from the resultant precipitate were isolated using ISOGEN (Nippon Gene, Osaka, Japan) and dissolved with 15 μL of RNase-free H_2_O.

### 2.7 miRNA microarray

Pulled-down RNAs from the λN/boxB system were analyzed with a miRNA microarray analysis contract service using Affymetrix GeneChip™ miRNA 4.0 Array (Filgen, Nagoya, Japan). Fold-change values were calculated by dividing the normalized expression value in starved cells by that in normal cells.

### 2.8 Reverse transcription-quantitative polymerase chain reaction (qPCR)

Using total, pulled-down, and isolated RNAs following photoactivatable ribonucleoside-enhanced crosslinking and immunoprecipitation (PAR-CLIP), miR-197-5p, miR-4530, miR-4734, miR-4745-5p, miR-6126, miR-6791-5p, miR-6798-5p, miR-6831-5p, *U6*, *HNRNPA1* mRNA, *HNRNPA1* mRNA 3ʹUTR, and *ACTB* mRNA levels were determined using qPCR. *U6* and *ACTB* served as internal standards. Detailed procedures have been described in our previous reports [18,25]. Primer sequences used in this experiment are listed in Supplementary Table 1.

### 2.9 miRNA and anti-miRNA transfection

Using the Dharmafect 1 reagent (Dharmacon, Lafayette, CO, USA), cells were transfected with biotinylated mature miR-4734, miR-4745-5p, miR-6126, miR-6798-5p (Fasmac, Kanagawa, Japan), biotinylated mature miR-4734 mutant (mt), miR-4745-5p mt, miR-6126 mt, miR-6798-5p mt (Fasmac), miRDIAN™ microRNA inhibitor of miR-4734, miR-4745-5p, miR-6126, miR-6798-5p (Dharmacon), unbiotinylated miR-4734 mt, miR-4745-5p mt, miR-6126 mt, miR-6798-5p mt (Fasmac), and universal negative control (Dharmacon) according to the manufacturer's protocol. Cells were used for downstream experiments 24 h after transfection.

### 2.10 Biotinylated miRNA pulldown and qPCR

Biotinylated miRNA-transfected cells were lysed with avidin-pulldown lysis buffer supplemented with RNaseOUT (Invitrogen) and pulled down with streptavidin-conjugated Dynabeads™ magnetic beads (Invitrogen) for 1 h at 25 °C. Thereafter, pulled-down RNAs were isolated and subjected to qPCR for *HNRNPA1* RNA mRNA 3ʹUTR, as described above (2.6. and 2.8.).

### 2.11 Luciferase assay using the HNRNPA1 mRNA 3ʹUTR

Cells were co-transfected with the internal pGL control vector and pRL-based reporter vectors at a molar ratio of 1:10 (total 550 ng vectors) using polyethylenimine MAX (Polysciences). After 48 h, relative luciferase activities were determined using the Dual-Luciferase Reporter Assay System (Promega) according to the manufacturer's protocol. Relative activities were calculated by dividing the pRL value by the pGL value.

### 2.12 PAR-CLIP/qPCR

Cells incubated under normal or serum/glucose-starved conditions were subjected to PAR-CLIP using mouse anti-Ago2 or mouse IgG (1:100 each; Wako). The detailed procedure is described in our previous report (18). RNAs in the Ago2-associated immunocomplex were isolated with ISOGEN (Nippon Gene) and dissolved with 15 μL of RNase-free H_2_O. miR-4734, miR-4745-5p, miR-6126, miR-6798-5p, and *HNRNPA1* mRNA 3ʹUTR levels were determined using qPCR.

### 2.13 Growth assays

Control and transfected cells were plated on 96-well plates (1 × 10^5^ cells/well or 2 × 10^5^ cells/well), preincubated for 16 h and then subsequently incubated in complete or serum/glucose-starved culture media for 24, 48, and 72 h. Viable cells were counted using the WST-8 assay (Dojindo, Kumamoto, Japan) following incubation.

### 2.14 Statistical analyses

All comparisons were performed using the two-tailed Student's *t*-test, and differences were considered statistically significant at *P* < 0.05.

## 3 Results

### 3.1 Identification of HNRNPA1 mRNA 3ʹUTR-bound miRNAs using the λN/boxB system

**Fig. 1:**
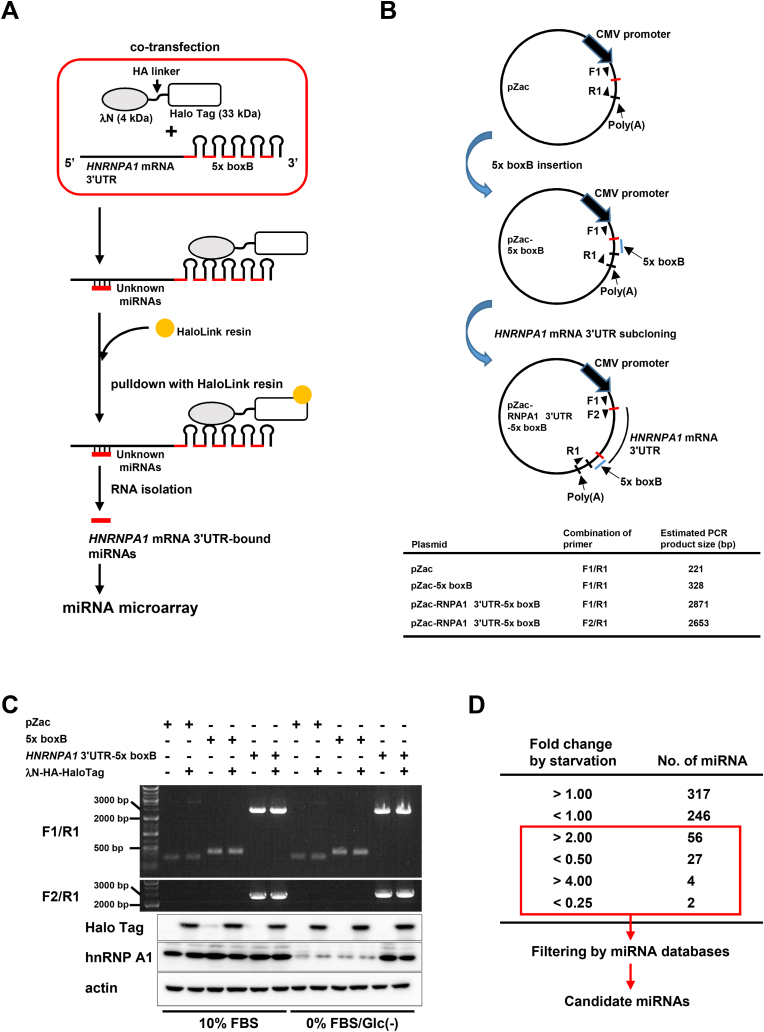
**Identification of serum/glucose starvation-driven miRNA targeting *HNRNPA1* mRNA 3ʹUTR.** A. Schematic flowchart of identification for *HNRNPA1* mRNA 3ʹUTR-binding miRNA identification the λN/boxB system combined with miRNA microarray. B. Construction chart for the 5 × boxB-fused RNA-expression vector. *Red*, multiple cloning site; *F1*, *F2*, and *R1*, positions of detection primers for RT-PCR. Estimated PCR product sizes are listed in bottom table. C. Representative pictures of RT-PCR and immunoblotting in HeLa cells co-transfected with the indicated vectors. Each combination of cotransfection was performed under normal and serum/glucose-starved conditions. D. Summary of miRNA microarray results and subsequent flowchart for candidate miRNA determination.

miRNAs bound to the *HNRNPA1* mRNA 3ʹUTR were isolated using the λN/boxB system (Fig. 1A). HeLa cells were co-transfected with a 5 × tandem boxB-fused RNA-expression vector and Halo tag-fused λN-expression vector. The 5 × boxB-fused RNA and Halo tag-fused λN interacted, forming a complex that can be isolated via pulldown using HaloLink resin. miRNAs bound to the 5 × boxB-fused *HNRNPA1* mRNA 3ʹUTR were isolated and identified via miRNA microarray analysis. Prior to identification, we performed a preliminary experiment to determine whether 5 × boxB-fused RNA and Halo Tag-fused λN peptides are expressed under serum/glucose-starved conditions. RT-PCR using vector-specific primers (between F1, F2, and R1; Fig. 1B) and immunoblotting confirmed the expression of 5 × boxB-fused RNA and Halo Tag-fused λN peptide under both normal and serum/glucose-starved conditions. Furthermore, immunoblotting revealed that serum/glucose starvation caused loss of hnRNP A1, except in 5 × boxB-fused *HNRNPA1* mRNA 3ʹUTR-expressing cells (Fig. 1C). This suggested that the 5 × boxB-fused *HNRNPA1* mRNA 3ʹUTR likely acted as a decoy for miRNAs targeting endogenous *HNRNPA1* mRNA 3ʹUTR. As in our previous report [18], serum/glucose-starvation did not induce cell death in HeLa cells. Additionally, no cytotoxicity induced by co-transfection was observed microscopically at least 48 h post-transfection (data not shown). An miRNA microarray was used to identify miRNAs preferentially binding to *HNRNPA1* mRNA 3ʹUTR under serum/glucose starvation. Microarray analysis revealed increased binding of 56 miRNAs to the *HNRNPA1* mRNA 3ʹUTR (52 miRNAs were >2.0-fold, and 4 were >4.0-fold; Fig. 1D). Conversely, decreased binding to the *HNRNPA1* mRNA 3ʹUTR was observed in 27 miRNAs (25 miRNAs were <0.5-fold and 2 were >0.25-fold; Fig. 1D and Supplementary Table 2). miRNAs with altered binding were subsequently filtered through public databases TargetScanHuman 7.1 (https://www.targetscan.org/vert_71/) and miRDB (https://mirdb.org/) to determine their potential binding to the *HNRNPA1* mRNA 3ʹUTR. Seventeen miRNAs were further identified as likely to bind to the *HNRNPA1* mRNA 3ʹUTR via their seed sequence (Table 1).

**Table 1:** Up- and downregulated miRNAs bound to *HNRNPA1* mRNA 3ʹUTR by serum/glucose starvation.

miRNA	fold change(stavation/10 % FBS)	predicted binding position at *HNRNPA1* mRNA 3ʹUTR (nt1-nt2543)	guide strand sequence (5′ to 3′)
hsa-miR-6126	4.064617404	982–988	GUGAAGGCCCGGCGGAGA
hsa-miR-1231	3.952873399	1664–1670	GUGUCUGGGCGGACAGCUGC
hsa-miR-6831-5p	3.128196597	1114–1120	UAGGUAGAGUGUGAGGAGGAGGUC
hsa-miR-197-5p	2.49929397	752–758	CGGGUAGAGAGGGCAGUGGGAGG
hsa-miR-4530	2.302630659	832–838	CCCAGCAGGACGGGAGCG
hsa-miR-4734	2.255311085	858–864	GCUGCGGGCUGCGGUCAGGGCG
hsa-miR-4745-5p	2.18571267	755–761	UGAGUGGGGCUCCCGGGACGGCG
hsa-miR-16-5p	2.231314498	107–113	UAGCAGCACGUAAAUAUUGGCG
hsa-miR-6791-5p	2.122347564	814–820	CCCCUGGGGCUGGGCAGGCGGA
hsa-miR-6798-5p	2.026211607	761–768	CCAGGGGGAUGGGCGAGCUUGGG
hsa-miR-3613-5p	0.114919173	60–66	UGUUGUACUUUUUUUUUUGUUC
hsa-miR-6750-5p	0.277104621	748–754	CAGGGAACAGCUGGGUGAGCUGCU
hsa-miR-1298-3p	0.281283325	624–630	CAUCUGGGCAACUGACUGAAC
hsa-miR-378h	0.31354268	1782-1788, 1830–1836	CAUCUGGGCAACUGACUGAAC
hsa-miR-3613-3p	0.341514826	263–269	ACAAAAAAAAAAGCCCAACCCUUC
hsa-miR-5189-3p	0.387056787	582–588	UGCCAACCGUCAGAGCCCAGA
hsa-miR-6808-3p	0.466657576	1103–1109	UGCCAACCGUCAGAGCCCAGA

### 3.2 Serum/glucose starvation-induced enhanced binding of HNRNPA1 mRNA 3ʹUTR with miR-4745-5p, miR-6126, and miR-6798-5p

**Fig. 2:**
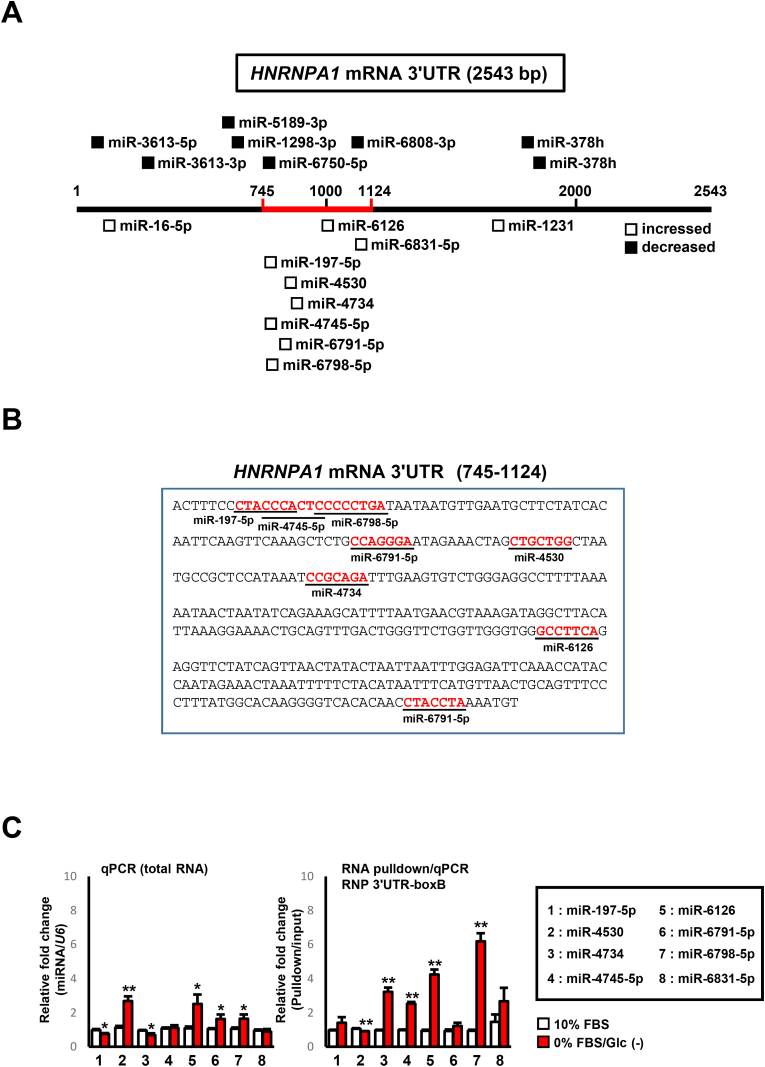
**Experimental screening of eight miRNA types for *HNRNPA1* mRNA 3ʹUTR binding.** A. Expected binding map of candidate miRNAs on *HNRNPA1* mRNA 3ʹUTR. The red highlighted section indicates the binding area of miRNAs where experiments were targeted. B. *HNRNPA1* mRNA 3ʹUTR sequences at nucleotides 745–1124 where candidate miRNAs bind via their seed sequences (red letters). C. Un-transfected or co-transfected (5 × boxB-fused *HNRNPA1* mRNA 3ʹUTR- and λN-Halo Tag-expression vectors) HeLa cells were incubated with complete or serum/glucose-starved culture media for 24 h, and total (un-transfected cells) and pulled-down (co-transfected cells) RNA were subjected to qPCR to detect the presence of miR-197-5p, miR-4530, miR-4734, miR-4745-5p, miR-6126, miR-6791-5p, miR-6798-5p, and miR-6831-5p. All experiments were performed in duplicate. *Columns*, mean values (*n* = 3); *bars*, SD; ∗P < 0.05 and ∗∗*P <* 0.01 using a two-tailed Student's *t*-test (comparison between the 10 % FBS and 0 % FBS/Glc (−) groups).

TargetScanHuman 7.1 was used to identify miRNA binding regions on the *HNRNPA1* mRNA 3ʹUTR. miRNAs with increased binding during starvation were concentrated in the *HNRNPA1* mRNA 3ʹUTR (8 of 10 types at nt745–nt1124), whereas miRNAs with decreased binding were almost equally distributed (Table 1 and Fig. 2A and B). Owing to the presence of “hot spots” (easily modified regions) in gene regulation, these eight miRNAs may play a central role in the serum/glucose starvation-induced regulation of hnRNP A1 expression. To examine whether the binding between the eight selected miRNAs and the *HNRNPA1* mRNA 3ʹUTR can be detected under other experimental conditions, qPCR analysis was performed using pulled-down RNAs from co-transfected cells in the λN/boxB system. This revealed that the increased binding between full-length *HNRNPA1* mRNA 3ʹUTR and endogenous miR-4734, miR-4745-5p, miR-6126, and miR-6798-5p was detected in serum/glucose-starved conditions (Fig. 2C). Total levels of miR-197-5p, miR-4734, miR-4745-5p, and miR-6831-5p did not increase under serum/glucose-starved conditions, whereas those of miR-4530, miR-6126, miR-6791-5p, and miR-6798-5p shows marginal increases. However the rates of elevation in miR-4734, miR-4745-5p, miR-6126, and miR-6798-5p were not higher than the results of qPCR using pulled-down RNAs (Fig. 2C). These results suggested that the binding between miR-4734, miR-4745-5p, miR-6126, and miR-6798-5p and *HNRNPA1* mRNA 3ʹUTR was confirmed.

**Fig. 3:**
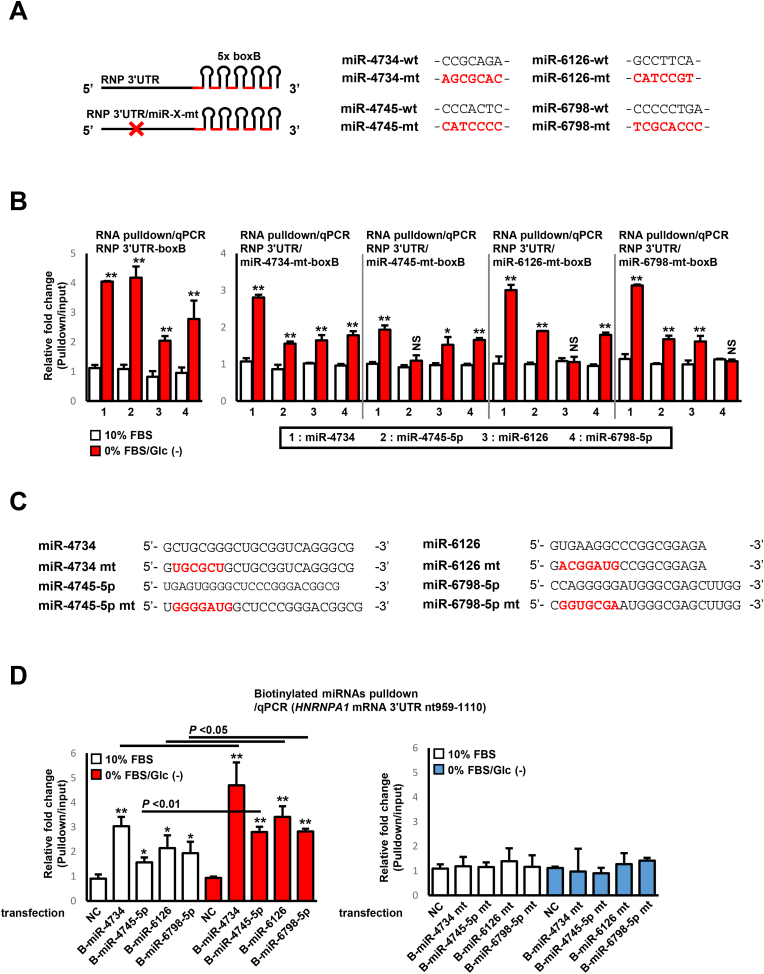
**miR-4734, miR-4745-5p, miR-6126, and miR-6798-5p are serum/glucose starvation-driven miRNAs for *HNRNPA1* mRNA 3ʹUTR.** A. Diagram of 5 × boxB-fused *HNRNPA1* mRNA 3ʹUTR and associated mutants. B. Cotransfected (5 × boxB-fused *HNRNPA1* mRNA 3ʹUTR-expression vector or 5 × boxB-fused mutant *HNRNPA1* mRNA 3ʹUTR-expression vectors, and λN-Halo Tag-expression vector) HeLa cells were incubated with complete or serum/glucose-starved culture media for 24 h and pulled-down RNAs were subjected to qPCR for the presence of miR-4734, miR-4745-5p, miR-6126, and miR-6798-5p. All experiments were performed in duplicate. *Columns*, mean values (*n* = 3); *bars*, SD; ∗∗*P <* 0.01 using a two-tailed Student's *t*-test (comparison between the 10 % FBS and 0 % FBS/Glc (−) groups); *NS*, not significant. C. The sequences of miR-4734, miR-4745-5p, miR-6126, and miR-6798-5p, and their mutants. Mutated regions are shown in red letters. D. HeLa cells were transfected with biotinylated miR-4734, miR-4745-5p, miR-6126, and miR-6798-5p, or their mutant counterparts. Transfected cells were lysed and pulled-down using streptavidin-conjugated magnetic beads. RNAs isolated from their precipitates were subjected to qPCR for the presence of *HNRNPA1* mRNA 3ʹUTR (nt959–1110). All experiments were performed in duplicate. *Columns*, mean values (*n* = 3); *bars*, SD; *NC*, universal negative control; *B*, biotinylated; ∗P < 0.05 and ∗∗P < 0.01 b y a two-tailed Student's *t*-test (comparison to NC-transfected group).

Next, we constructed RNA-expression vectors expressing 5 × boxB-fused *HNRNPA1* mRNA 3ʹUTR bearing a mutation that interrupts binding to miR-4734, miR-4745-5p, miR-6126, and miR-6798-5p (as indicated in Fig. 3A). qPCR analysis using these vectors revealed that the mutation at the miR-4745-5p, miR-6126, and miR-6798-5p binding sites in the *HNRNPA1* mRNA 3ʹUTR lacked binding ability to corresponding miRNAs under serum/glucose-starved condition, whereas no effects were observed to other types of miRNAs (Fig. 3B). A binding assay was performed using synthesized miRNAs and their mutants, with biotin conjugated to the 3ʹ-terminus (Fig. 3C). Cells were transfected with these synthesized oligos, lysed, and the complexes were pulled-down with streptavidin-conjugated magnetic beads. RNAs from each precipitant were subjected to qPCR for endogenous *HNRNPA1* mRNA 3ʹUTR. As shown in Fig. 3D, all transfected miRNAs were bound to endogenous *HNRNPA1* mRNA 3ʹUTR, and serum/glucose starvation potentiated their binding capacity in all miRNA-transfected groups. However, transfection with mutated miRNAs showed no binding, both in normal or serum/glucose-starved conditions. These results suggested that the binding of *HNRNPA1* mRNA 3ʹUTR to miR-4745-5p, miR-6126, and miR-6798-5p is driven under serum/glucose-starved conditions.

### 3.3 miR-4745-5p and miR-6798-5p targets hnRNP A1 protein level via its 3ʹUTR

**Fig. 4:**
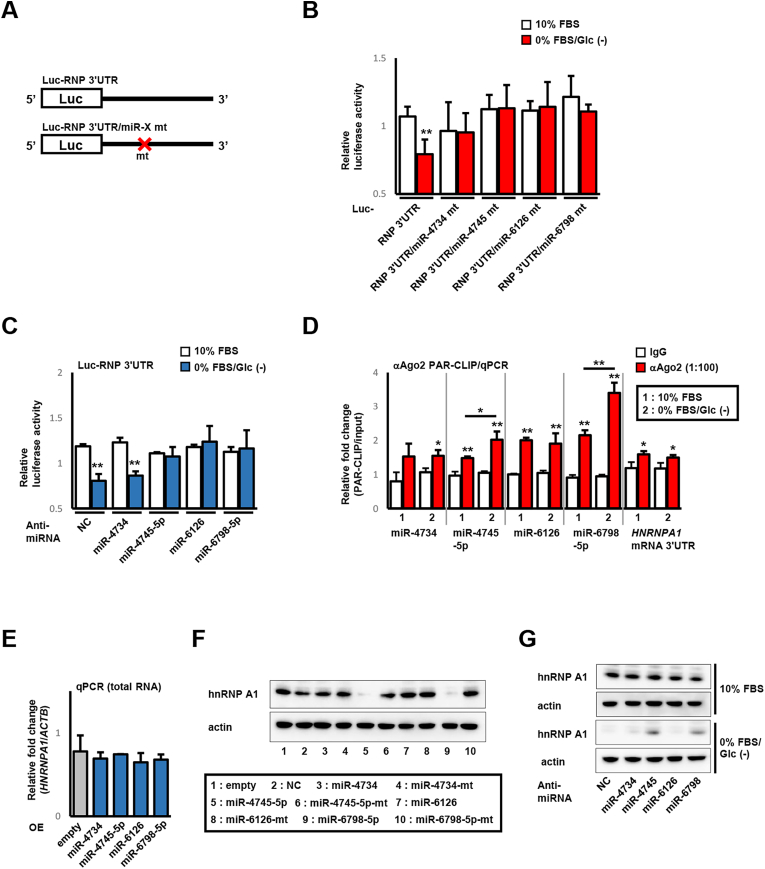
**Serum/glucose starvation-induced hnRNP A1 repression is mediated by miR-4745-5p and miR-6798-5p. A**. Diagram of the Luc-RNP 3ʹUTR and its mutant. B. HeLa cells were co-transfected with internal pGL control and reporter vectors as indicated. Sequences of mutation are the same as shown in Fig. 3A. After incubation with complete or serum/glucose-starved culture media for 24 h, relative luciferase activities were calculated by dividing the pRL value by the pGL value. All experiments were performed in duplicate. *Columns*, mean values (*n* = 3); *bars*, SD; ∗∗*P <* 0.01 using a two-tailed Student's *t*-test (comparison between the 10 % FBS and 0 % FBS/Glc (−) groups). C. HeLa cells were transfected with anti-miRNA for a universal negative control, miR-4734, miR-4745-5p, miR-6126, and miR-6798-5p. After 24 h, cells were further co-transfected with an internal pGL control vector and the RNP 3ʹUTR reporter vector. After incubation with complete or serum/glucose-starved culture media for 24 h, relative luciferase activities were calculated as described above. All experiments were performed in triplicate. *Columns*, mean values (*n* = 3); *bars*, SD; *NC*, universal negative control, ∗P < 0.05 and ∗∗*P <* 0.01 using a two-tailed Student's *t*-test (comparison between the 10 % FBS and 0 % FBS/Glc (−) groups). D. Lysates from normal or serum/glucose-starved cells were treated with the anti-Ago2 antibody or isotype IgG and precipitated with protein G-conjugated magnetic beads. RNAs isolated from their immune precipitates were subjected to qPCR for the presence of miR-4734, miR-4745-5p, miR-6126, miR-6798-5p, and *HNRNPA1* mRNA 3ʹUTR. All experiments were performed in duplicate. *Columns*, mean values (*n* = 3); *bars*, SD; ∗P < 0.05 and ∗∗*P <* 0.01 using a two-tailed Student's *t*-test (comparison between the IgG- and anti-Ago2–treated groups, except the comparison indicated by the line). E. HeLa cells were transfected with empty- or miRNA-expression vectors for miR-4734, miR-4745-5p, miR-6126, and miR-6798-5p for 48 h. Total RNA from these cells was subjected to qPCR for *HNRNPA1* mRNA. All experiments were performed in triplicate. *Columns*, mean values (*n* = 3); *bars*, SD; *OE*, overexpression. F. HeLa cells were transfected with empty- or miRNA-expression vectors for miR-4734, miR-4745-5p, miR-6126, miR-6798-5p, along with a universal negative control, and mutated miR-4734, miR-4745-5p, miR-6126, and miR-6798-5p for 48 h. Mutation sequences are the same as those in Fig. 3C. Lysates from these cells were used in immunoblotting for hnRNP A1. Pictures are representative of four independent experiments. *OE*, overexpression; *NC*, universal negative control. G. HeLa cells were transfected with an anti-miRNA for miR-4734, miR-4745-5p, miR-6126, and miR-6798-5p. After 24 h, cells were incubated with complete or serum/glucose-starved culture media for 24 h, lysed, and immunoblotted for hnRNP A1. Pictures are representative of two independent experiments. *NC*, universal negative control.

We performed a luciferase reporter assay, qPCR following PAR-CLIP, qPCR, and immunoblotting to confirm and identify serum/glucose starvation-driven functional miRNAs. A luciferase reporter vector was used with *HNRNPA1* mRNA 3ʹUTR-based genes fused downstream of the luciferase gene, and reporter activities were examined under normal or serum/glucose-starved conditions. The tested genes included the *HNRNPA1* mRNA 3ʹUTR (Luc-RNP 3ʹUTR) and mutants that could not bind miR-4734, miR-4745-5p, miR-6126, or miR-6798-5p (Fig. 3A) (Luc-RNP 3ʹUTR/miR-4734 mt, Luc-RNP 3ʹUTR/miR-4745 mt, Luc-RNP 3ʹUTR/miR-6126 mt, and Luc-RNP 3ʹUTR/miR-6798 mt, respectively; Fig. 4A). As expected, the reporter activity of Luc-RNP 3ʹUTR significantly decreased under serum/glucose starvation. The reporter activities of all mutant reporters showed no alteration compared to Luc-RNP 3ʹUTR under normal conditions. Noteworthy, those of all mutant reporters also did not decrease under serum/glucose-starved conditions (without any significance, Fig. 4B). Next, we tested effect of treatment of anti-miRNA for miR-4734, miR-4745-5p, miR-6126, and miR-6798-5p on the reporter activity of Luc-RNP 3ʹUTR. The reporter activities of all anti-miRNA-treated cells showed no alteration compared to universal negative control-treated cells under normal conditions. The reporter activity was significantly decreased under serum/glucose starvation in universal negative control-treated cells. In contrast, treatment of anti-miRNA for miR-4745-5p, miR-6126, or miR-6798-5p apparently restored decrease of the reporter activity, whereas that of anti-miRNA for miR-4734 did not (Fig. 4C). To assess whether their miRNAs were preferentially recruited to the RNA-induced silencing complex (RISC) under serum/glucose-starved conditions, we performed a PAR-CLIP experiment using the anti-Ago2 antibody. Regardless of serum/glucose supplementation, miR-4745, miR-4745-5p, miR-6126, miR-6798-5p, and *HNRNPA1* mRNA 3ʹUTR (expected binding partner of examined miRNAs) were detected in RISC. Notably, serum/glucose starvation significantly increased miR-4745-5p and miR-6798-5p levels in RISCs (Fig. 3D). Finally, we examined whether miR-4745-5p and miR-6798-5p affect the endogenous hnRNP A1 expression level. The transient overexpression of miR-4734, miR-4745-5p, miR-6126, and miR-6798-5p showed no change in mRNA levels (Fig. 4E), whereas an evident decrease in hnRNP A1 protein level was observed in miR-4745-5p- and miR-6798-5p-overexpressed cells, and a marginal decrease was found in miR-6126-overexpressed cells (Fig. 4F). In contrast, the transient transfection of mutated miRNAs did not alter the hnRNP A1 protein level (Fig. 4F). An experiment using anti-miRNAs further showed that inhibition of cellular miRNA by anti-miRNA for miR-4745-5p and miR-6798-5p attenuated serum/glucose starvation-induced loss of endogenous hnRNP A1, whereas these effects were not observed under normal conditions (Fig. 4G). These results indicated that miR-4745-5p and miR-6798-5p serve as serum/glucose starvation-driven miRNAs for hnRNP A1 protein expression.

### 3.4 hnRNP A1-dependent effect of miR-4745-5p and miR-6798-5p on cellular growth in HeLa cells

**Fig. 5:**
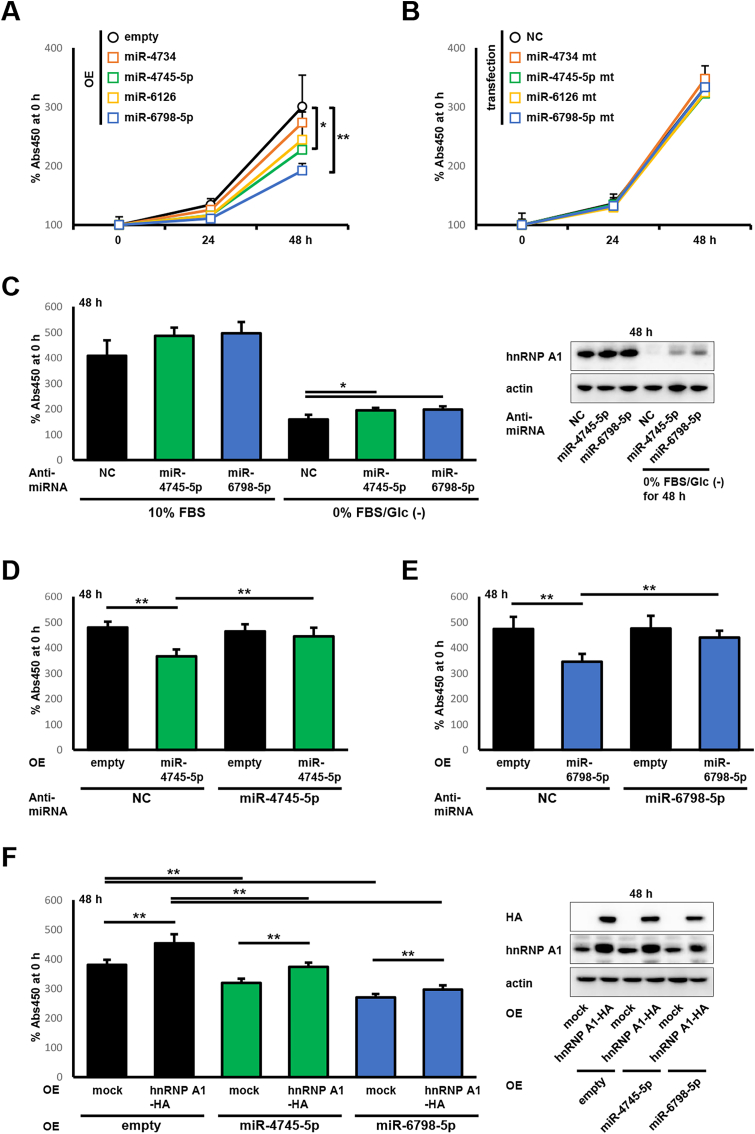
**miR-4745-5p and miR-6798-5p suppress cell growth in an hnRNP A1-dependent manner.** A. HeLa cells transfected with empty- or miRNA-expression vectors for miR-4734, miR-4745-5p, miR-6126, and miR-6798-5p were incubated in complete media and cell growth was measured using the WST-8 assay. The A_450_ value at 0 h was designated as 100 % in the WST-8 assay, and the relative percentages at 24 and 48 h are shown. All experiments were performed in triplicate. *Symbols*, mean percentages (*n* = 6); *bars*, SD; *OE*, overexpression; ∗P < 0.05 and ∗∗*P <* 0.01 using a two-tailed Student's *t*-test. B. HeLa cells transfected with universal negative control, mutated miR-4734, miR-4745-5p, miR-6126, and miR-6798-5p were subjected to the WST-8 assay as in Fig. 5A. All experiments were performed in triplicate. *Symbols*, mean percentages (*n* = 6); *bars*, SD; *NC*, universal negative control. C. HeLa cells transfected with the universal negative control, an anti-miRNA for miR-4745-5p, and miR-6798-5p were incubated in complete or serum/glucose-starved culture media. Cell growth after 48 h was measured using the WST-8 assay. The A_450_ value at 0 h was designated as 100 % in WST-8 assay, and the relative percentages at 48 h are shown. All experiments were performed in duplicate. *Columns*, mean percentages (*n* = 6); *bars*, SD; *NC*, universal negative control; ∗*P <* 0.05 using a two-tailed Student's *t*-test. In parallel, these cells were subjected to immunoblotting for hnRNP A1. Pictures are representative of two independent experiments. *NC*, universal negative control. D-E. HeLa cells co-transfected with empty vector plus universal negative control, anti-miRNA for miR-4745-5p, or anti-miRNA for miR-6798-5p, miRNA-expression vectors for miR-4745-5p plus universal negative control or anti-miRNA for miR-4745-5p, and miRNA-expression vectors for miR-6798-5p plus universal negative control or anti-miRNA for miR-6798-5p were incubated in complete media, and cell growth after 48 h was measured using the WST-8 assay. The A_450_ value at 0 h was designated as 100 % in the WST-8 assay, and the relative percentages at 48 h are shown. All experiments were performed in duplicate. *Columns*, mean percentages (*n* = 6); *bars*, SD; *NC*, universal negative control; *OE*, overexpression; ∗∗*P <* 0.01 using a two-tailed Student's *t*-test. F. HeLa cells co-transfected with the empty vector plus mock or hnRNP A1-HA expression vector, miRNA-expression vectors for miR-4745-5p plus mock or hnRNP A1-HA expression vector, and miRNA-expression vectors for miR-6798-5p plus mock or hnRNP A1-HA expression vector were incubated in complete media, and cell growth was measured after 48 h using the WST-8 assay. The A_450_ value at 0 h was designated as 100 % in the WST-8 assay, and the relative percentages at 48 h are shown. All experiments were performed in triplicate. *Columns*, mean percentages (*n* = 6); *bars*, SD; *OE*, overexpression; ∗P < 0.05 and ∗∗*P <* 0.01 using a two-tailed Student's *t*-test. In parallel, these cells were subjected to immunoblotting for HA and hnRNP A1. Pictures are representative of two independent experiments. *OE*, overexpression.

To clarify whether miR-4745-5p and miR-6798-5p are functional miRNAs for cellular growth via hnRNP A1 repression, five types of transiently transfected cells overexpressing miR-4734, miR-4745-5p, miR-6126, and miR-6798-5p were subjected to a WST-8 assay. As shown in Fig. 5A, the increase in cell number at 48 h after incubation was significantly lower in miR-4745-5p- and miR-6798-5p-overexpressing cells than that in empty vector-transfected cells. However, miR-4734- and miR-6126- overexpressing cells showed no difference in cell number increase. In contrast, cells transfected with miRNA mutants whose sequences are shown in Fig. 3C, exhibited no change in growth rate (Fig. 5B). Cells were subsequently transfected with anti-miRNA for miR-4745-5p and miR-6798-5p and incubated in either complete or serum/glucose-starved media. Under incubation in complete media, cell number increase rates at 48 h after incubation were marginally higher (yet not significantly) in cells transfected with anti-miRNA for miR-4745-5p and miR-6798-5p. Consequently, transfection with anti-miRNA for miR-4745-5p and miR-6798-5p significantly promoted cell number increase at 48 h serum/glucose-starved conditions (Fig. 5C). Immunoblotting confirmed that the expression status of hnRNP A1 was consistent with Fig. 4G (Fig. 5C), suggesting that the growth promotion by anti-miRNA for miR-4745-5p and miR-6798-5p is due to the restoration of hnRNP A1. In rescue experiments, co-transfection with miR-4745-5p and miR-6798-5p expression vectors and anti-miRNA for miR-4745-5p and miR-6798-5p was performed. In empty vector-transfected HeLa cells, anti-miRNA for miR-4745-5p and miR-6798-5p had no effect on cell number increase rates at 48 h. HeLa cells transfected with anti-miRNA negative control plus miR-4745-5p and miR-6798-5p expression vectors were showed a significant decrease in cell number increase at 48 h, whereas transfection with anti-miRNA for miR-4745-5p and miR-6798-5p plus expression vectors of miR-4745-5p and miR-6798-5p significantly restored cell number increase at 48 h (Fig. 5D and E). Finally, we examined the effect of hnRNP A1 overexpression on cellular growth in miR-4745-5p- and miR-6798-5p-overexpressing HeLa cells. As in previous results, miR-4745-5p- and miR-6798-5p-overexpressing HeLa cells showed a significant decrease in cell number increase at 48 h than in empty vector-transfected HeLa cells. When hnRNP A1-HA were overexpressed, the cell number increase at 48 h was significantly higher in all cell lines (those transfected with an empty vector, miR-4745-5p-expression vector, and miR-6798-5p-expression vector). Additionally, immunoblotting indicated successful expression of hnRNP A1-HA (Fig. 5F). These results indicated that hnRNP A1 is partially involved in growth suppression by miR-4745-5p and miR-6798-5p.

## 4 Discussion

In bacteriophage λ, transcriptional termination is suppressed via a multifactor complex that includes the N peptide and boxB sequence at the *nut* site, facilitating in bypassing the downstream terminator region [26,27]. Our λN/boxB system leverages the specific interaction between the λ phage-derived N peptide and the boxB RNA sequence [[28], [29], [30]], an interaction that can be used to tether λN-fused transcription factors to the boxB-inserted promoter region [[31], [32], [33]]. However, we considered that this strong, specific interaction could also serve us a tool for detecting molecules bound to “desired RNA” sequences. Hence, to establish a novel identification method for RNA-binding factors using this interaction, we adapted a pulldown-based procedure, allowing for isolation, purification, and/or concentration steps necessary for comprehensive analysis, such as next-generation sequencing or microarrays. Notably, serum/glucose starvation reduces hnRNP A1 protein levels without altering mRNA levels, suggesting posttranscriptional regulation involving miRNAs [18]. This background motivated us to determine the serum/glucose starvation-driven miRNAs for hnRNP A1 by using the λN/boxB system.

Using co-transfection with Halo tag-fused λN- and 5 × boxB-fused *HNRNPA1* mRNA 3ʹUTR-expression vectors and subsequent pulldown, we performed miRNA microarray analysis to identify serum/glucose starvation-driven miRNAs for hnRNP A1. Eight candidate miRNAs were initially selected based on information from miRNA databases. Subsequent binding analyses with intact or mutant constructs and biotinylated miRNAs narrowed down the candidates to miR-4745-5p, miR-6126, and miR-6798-5p. Reporter assays, qPCR, PAR-CLIP/qPCR, and immunoblotting were conducted to identify functional miRNAs that regulate hnRNP A1 protein expression. These assays revealed that miR-4745-5p and miR-6798-5p act as serum/glucose starvation-driven functional miRNAs that downregulate protein levels. However, pretreatment with anti-miRNA for these candidates only partially recovered hnRNP A1 protein loss by serum/glucose starvation, suggesting that miR-4745-5p and miR-6798-5p also targets other genes and *HNRNPA1*, diluting their specific effect on *HNRNPA1* mRNA.

As several reports indicate that hnRNP A1 promotes cancer cell growth [12,14,34], we tested whether miR-4745-5p and miR-6798-5p for effects on cancer cell growth via an hnRNP A1-dependent manner. As expected, miR-4745-5p and miR-6798-5p overexpression significantly reduced cellular growth, whereas mutated versions did not. Anti-miRNAs for miR-4745-5p and miR-6798-5p increased cellular growth under serum/glucose-starved conditions and reversed their growth suppressing effects. Furthermore, re-expression of hnRNP A1 also reversed miR-4745-5p- and miR-6798-5p-mediated growth suppression, suggesting that they regulate hnRNP A1-associated cellular growth. Using TargetScanHuman 7.1, nine growth-related genes (*IGF1*, *IGF1R*, *IGF2R*, *IGFBP4*, *IGFBP5*, *IGF2BP1*, *FGF11*, *FGF19*, and *TGFA*) were identified as common targets of miR-4745-5p and miR-6798-5p [[35], [36], [37], [38], [39], [40], [41], [42]]. The anti-miRNA's effect may involve a combination of influence on *HNRNPA1* gene and other target genes. This hypothesis is credible as anti-miRNAs incompletely restored hnRNP A1 protein expression, suggesting that anti-miRNAs also acted on additional gene targets. Currently, only four studies on miR-4745-5p and miR-6798-5p have been published [[43], [44], [45], [46]], emphasizing the need for detailed investigation, especially in cancer cells. Moreover, the cellular status-dependent mechanism of miRNA recruitment to the mRNA 3ʹUTR region is unclear and warrants further investigation to clarify how serum/glucose starvation enhances binding between specific miRNAs and *HNRNPA1* mRNA 3ʹUTR.

The λN/boxB system was used to identify prey miRNAs using the boxB-fused *HNRNPA1* mRNA 3ʹUTR as bait. This system can be applied in various of detection methods to identify molecules specifically bound to a “desired RNA” sequence. For example of use, it is being used to detect novel proteins bound to the *HNRNPA1* mRNA 3ʹUTR by the λN/boxB system via silver staining, SDS-PAGE, and liquid chromatography/mass spectrometry. Furthermore, the λN/boxB system is being developed to identify regulatory factors for pre-mRNA splicing, using a pre-mRNA sequence across the intron–exon boundary region as bait. This system is also applicable for binding analysis of “desired RNA” or for identifying binding motifs of RNA-interacting molecules. Another advantage of the λN/boxB system is that its results likely reflect intracellular reactions owing to its basis in cellular co-expression. This feature of the λN/boxB system helps minimize artificial results, making it a reliable method.

## 5 Conclusion

The λN/boxB system enabled the identification of miR-4745-5p and miR-6798-5p as serum/glucose starvation-driven miRNAs targeting hnRNP A1. Detailed functional analysis of miR-4745-5p and miR-6798-5p is crucial for understanding the biological significance of hnRNP A1 protein depletion in serum/glucose-deficient lesions, such as deep tumor masses. Furthermore, the λN/boxB system, may, in principle, use any “desired RNA” sequence as bait, making it a valuable tool for analyzing RNA-interacting factors, regardless of whether they are DNA, RNA, or proteins. Understanding how serum/glucose starvation induces specific miRNA activation is a critical area for future research.

## CRediT authorship contribution statement

**Tetsuyuki Takahashi:** Writing – original draft, Validation, Supervision, Methodology, Investigation, Funding acquisition, Data curation, Conceptualization. **Mai Funamura:** Validation, Data curation. **Shun Wakai:** Validation, Data curation. **Takao Hijikata:** Writing – review & editing, Supervision, Conceptualization.

## Data availability statement

The data that support the findings of this study are available upon request from the corresponding author.

## Funding

This study was supported by JSPS KAKENHI (grant number JP21K06538) and a grant from 10.13039/100019640Musashino University to TT.

## Declaration of competing interest

The authors have declared that no competing interests exist.
