# Supplementary material for: Serum/glucose starvation enhances binding of miR-4745-5p and miR-6798-5p to HNRNPA1 mRNA 3ʹUTR: A novel method to identify miRNAs binding to mRNA 3ʹUTR using λN peptide-boxB sequence
Source: Noncoding RNA Res. 2025 Jan 7;11:188–99. doi: 10.1016/j.ncrna.2025.01.001 (PMC11786793; doi:10.1016/j.ncrna.2025.01.001)
Supplement: Multimedia component 1 [file mmc1.docx]

**Supplementary information**

**Serum/glucose starvation enhances binding of miR-4745-5p and miR-6798-5p to *HNRNPA1* mRNA 3ʹUTR: A novel method to identify miRNAs binding to mRNA 3ʹUTR using λN peptide-boxB sequence**

**Supplementary Methods**

**Supplementary Methods**

**Plasmid construction**

Successful construction of all vectors was confirmed by using direct sequencing with a 3500 genetic analyzer (Applied Biosystems, Foster City, CA, USA). Primer sequences are described in the Supplementary Table S1.

***BoxB- and boxB-HNRNPA1 mRNA 3ʹUTR expression plasmids***

The 5× boxB sequence and *HNRNPA1* mRNA 3ʹUTR-5× boxB were inserted into the mammalian expression vector pZac [1]. Insertion of the 5× boxB sequence was performed using the KOD-neo mutagenesis kit (TOYOBO, Osaka, Japan). *HNRNPA1* mRNA 3ʹUTR cDNA was synthesized by reverse transcription from HeLa-derived total RNA, and then subcloned into the above pZac-5× boxB vector to give pZac-RNP3ʹUTR-5× boxB.

Using pZac-RNP3ʹUTR-5× boxB, mutation of binding sites for miR-4734, miR-4745-5p, miR-6126, and miR6798-5p (Fig. 3A) were conducted using the KOD-neo mutagenesis kit (TOYOBO). Subsequent plasmids were designated as pZac-RNP3ʹUTR/miR-4734-mt-5 × boxB, pZac-RNP3ʹUTR/miR-4745-mt-5 × boxB, pZac-RNP3ʹUTR/miR-6126-mt-5 × boxB, and pZac-RNP3ʹUTR/miR-6798-mt-5 × boxB, respectively.

***λN peptide-fused Halo tag expression plasmid***

The coding sequence of the HA-tagged λN peptide was inserted into the Halo-tag expression plasmid pFC15K (Promega) using the KOD-neo mutagenesis kit (TOYOBO). The HA coding sequence was used as a linker between the λN peptide and Halo-tag. This plasmid was designed as pFC-λN-HA-Halo.

***Luciferase reporter plasmids***

Using pRL-CMV (Promega), *HNRNPA1* mRNA 3ʹUTR obtained from HeLa cells was subcloned downstream of the luciferase gene to generate pRL-Luc-RNP3ʹUTR. Using pRL-Luc-RNP3ʹUTR, mutation of binding site for miR-4734, miR-4745-5p, miR-6126, and miR6798-5p (Fig. 4A) were conducted using the KOD-neo mutagenesis kit (TOYOBO). Subsequent plasmids were designated as pRL-Luc-RNP3ʹUTR/miR-4734 mt, pRL-Luc-RNP3ʹUTR/miR-4745 mt, pRL-Luc-RNP3ʹUTR/miR-6126 mt, and pRL-Luc-RNP3ʹUTR/miR-6798 mt, respectively.

***miRNA expression plasmids***

The retroviral expression plasmid pSIREN-RetroQ (pSRQ; Takara Bio, Shiga, Japan) was for miRNA-expression plasmids. Using the KOD-neo mutagenesis kit (TOYOBO), guide sequences of miR-4734, miR-4745-5p, miR-6126, and miR6798-5p were inserted into the multicloning site of pSRQ. Subsequent plasmids were designated as pSRQ-miR4734, pSRQ-miR4745, pSRQ-miR6126, and pSRQ-miR6798, respectively.

***hnRNP A1-HA expression plasmid***

The mammalian hnRNP A1-HA expression plasmid pQCXIP-hnRNP A1-HA has been previously constructed [2].

**Supplementary References**

[1] T. Takahashi, H. Ichikawa, Y. Okayama, M. Seki, T. Hijikata, SV40 miR-S1 and cellular miR-1266 sequester each other from their targets, enhancing telomerase activity and viral expression, Non-coding RNA 8 (2022) 57, https://doi.org/[10.3390/ncrna8040057](https://doi.org/10.3390/ncrna8040057).

[2] T. Takahashi, Y. Ando, H. Ichikawa, K. Tsuneyama, T. Hijitaka, Serum/glucose starvation strikingly reduces heterogenous nuclear ribonucleoprotein A1 protein and its target, cyclin D1, FEBS J. 290 (2023) 4126–4144, https://doi.org/[10.1111/febs.16802](https://doi.org/10.1111/febs.16802).
